# Longer Duration of SARS-CoV-2 Infection in a Case of Mild COVID-19 With Weak Production of the Specific IgM and IgG Antibodies

**DOI:** 10.3389/fimmu.2020.01936

**Published:** 2020-08-07

**Authors:** Xuemin Guo, Lizhu Zeng, Zhen Huang, Yongjun He, Zhuojin Zhang, Zhixiong Zhong

**Affiliations:** ^1^Meizhou People's Hospital, Meizhou, China; ^2^Guangdong Provincial Key Laboratory of Precision Medicine and Clinical Translation Research of Hakka Population, Meizhou, China

**Keywords:** mild COVID-19, SARS-CoV-2, persistent infection, virus-specific antibody response, case report

## Abstract

**Background:** The relationship between SARS-CoV-2-carrying time and specific antibody production has not yet been reported in re-admitted COVID-19 patients. We reported a case of mild COVID-19 with long virus-carrying time, weak production of virus-specific IgG and IgM antibodies, and recurrence of positive SARS-CoV-2 RNA in stool specimens after discharge.

**Case Presentation:** A 27-year-old male was diagnosed as COVID-19 after returning to Meizhou from Wuhan. Despite extremely mild symptoms, the patient was hospitalized for 24 days because of persistent positive SARS-CoV-2 RNA detection. Three days after recovery discharge, he was hospitalized again for 7 days due to a recurrence of the positive SARS-CoV-2 RNA result, while in a good physical condition. Serological assay, using a fluorescent immunochromatography detection kit specific to SARS-CoV-2, showed that SARS-CoV-2-specific IgM antibodies were undetectable and IgG antibodies were very low on day 8 after onset; both of the antibodies seemingly reached top concentrations on day 15 (just a 6-fold increase of the IgG titer), and then decreased, remaining relatively stable from day 25 after onset until discharge. The production of the IgM and IgG targeting SARS-CoV-2 in this very mild case was much lower than that in a severe case of COVID-19 during the same hospitalizing period, and the latter was used as a control.

**Conclusion:** Mild COVID-19 patients could carry SARS-CoV-2 for a long time, which may be related to the weak production of the virus-specific IgG and IgM. Recurrence of positive SARS-CoV-2 RNA could occur in mild COVID-19 possibly due to intermittent virus shedding, so strict quarantine and health surveillance should be taken for all discharged COVID-19 patients to prevent a potential virus spread.

## Introduction

In late December 2019, coronavirus disease 2019 (COVID-19) broke out in Wuhan, China, and then spread rapidly throughout the country, followed by a worldwide epidemic (1). In addition to a small number of asymptomatic infections, the clinical classification of COVID-19 includes mild cases, moderate cases, severe cases, and critical cases (2). About 80% of laboratory-confirmed patients had a mild or moderate disease, 13.8% were severe, and 6.1% were critical, and the crude mortality rate was about 3.8% (3).

Severe acute respiratory syndrome coronavirus 2 (SARS-CoV-2) is highly contagious, and clinical symptoms primarily manifest as a fever, dry cough, fatigue, shortness of breath, and multiple ground-glass opacities in both lungs (2–5). The positive nucleic acid test results of upper and lower respiratory tract specimens and stool specimens are the basis of COVID-19 laboratory confirmation; two consecutively negative results taken 24 h apart is an essential criterion for discharge. From the onset of symptoms to clinical recovery, the median time is about 2 weeks for mild cases, and 3–6 weeks for severe and critical cases (2, 3). According to reports, some discharged patients have been readmitted for recurrence of positive SARS-CoV-2 RNA (6–8). Viral infection can stimulate the immune system to produce specific antibodies. It has been predicted that the patient's body produces SARS-CoV-2-specific IgM antibodies about 3–5 days after onset, followed by the production of the specific IgG antibodies; the IgG titer increases at least 4-fold during the recovery stage, relative to the acute stage (2).

This report presents a very mild COVID-19 case with persistent SARS-CoV-2 infection lasting at least 30 days and weak production of the antibodies specific to SARS-CoV-2. Noticeably, this patient was hospitalized again due to recurrence of positive SARS-CoV-2 RNA; his virus-specific IgM was undetectable on day 8 after onset; the amount of IgG was just slightly greater than the cutoff value on day 8 after onset and peaked on day 15 with about a 6-fold increase.

## Case Presentation

A 27-year-old man who had been living in Wuhan returned to Meizhou on January 20, 2020. He developed a low fever (38°C), an occasional cough, and mild chills on the evening of January 24. The SARS-CoV-2 RNA test result of his throat swab specimens was positive, so he was confirmed to have COVID-19 and was admitted to our hospital on January 26. On admission, a physical examination showed: (1) low fever (37.2°C) and occasional dry cough; (2) mood, appetite, and sleep were merely acceptable; (3) heart rate: 80 beats/min, breathing rate: 20 times/ min, and blood pressure: 120/68 mmHg. Chest CT scan revealed one instance of dorsal cord inflammation in each of the upper and lower lobes of the right lung. Laboratory tests showed (1) slightly low arterial oxygen saturation (93.6%); (2) normal white blood cell count (4.0 × 10^9^/L), lymphocyte count (1.5 × 10^9^/L), and CRP value (2.36 mg/L); (3) normal liver and kidney function indicator values; (4) absence of other viral infections, including HIV, influenza A and B virus, respiratory syncytial virus, adenovirus, *Chlamydia pneumoniae, Legionella pneumophila*, and parainfluenza virus.

**Figure 1:**
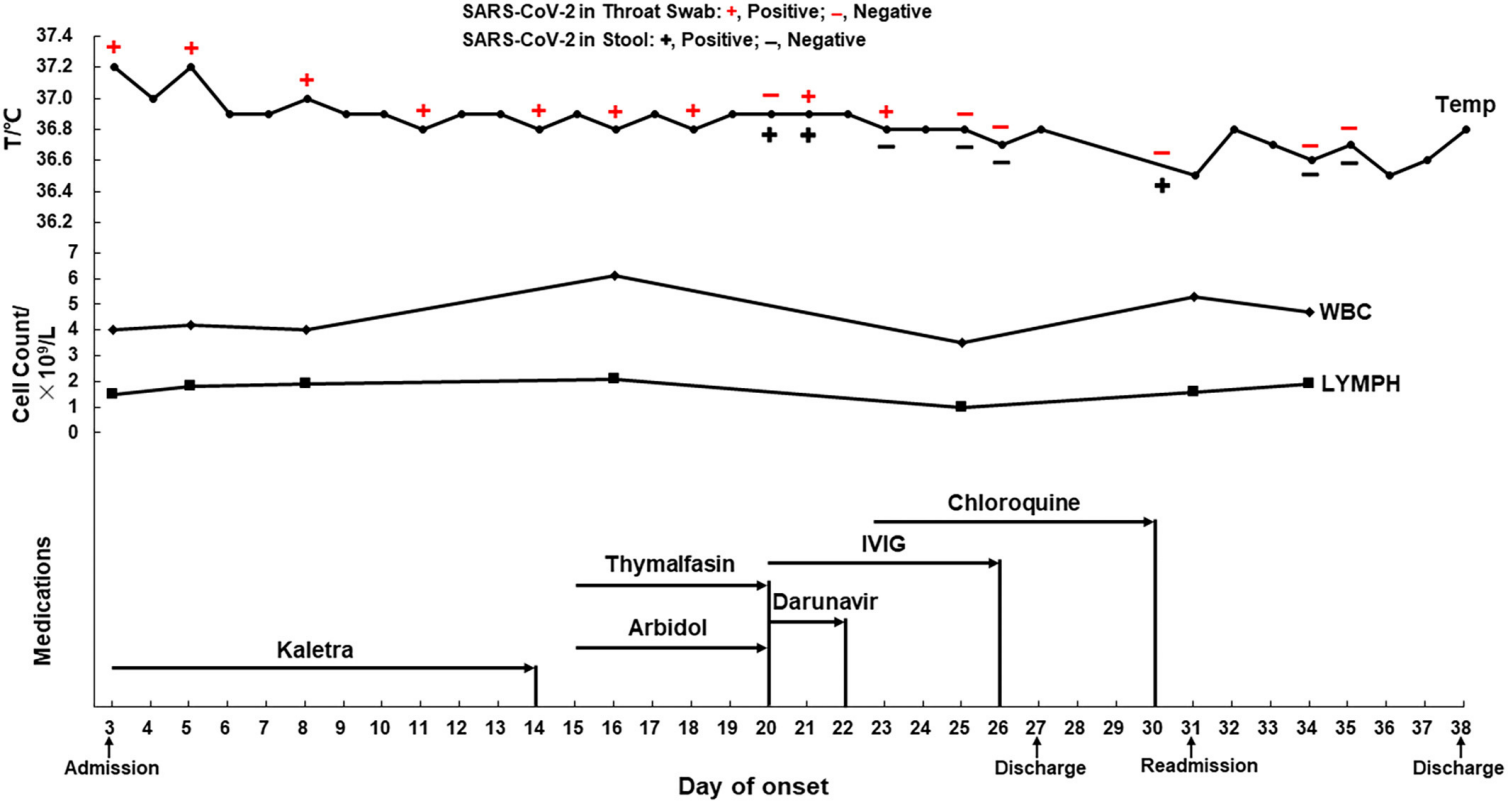
The dynamics of the throat swab and stool sample test of SARS-CoV-2 RNA, body temperature, blood cell counts, and medications of a mild COVID-19 during hospitalization. Antiviral medication: Kaletra, Arbidol, Darunavir, and Chloroquine. Supplementary medication: Thymalfasin and IVIG. IVIG, intravenous immunoglobulin; WBC, white blood cell count (3.5–9.5 × 10^9^/L); LYMPH, lymphocytes (1.1–3.2 × 10^9^/L).

After admission, the patient was given oxygen through a nasal catheter and nutritional support treatment. During hospitalization, the patient experienced an occasional cough, but his temperature and white blood cell and lymphocyte counts were normal (Figure 1, upper panel and middle panel, respectively), and his vital signs were stable. He was given medications that had been predicted to have anti-SARS-CoV-2 activity, specifically Kaletra, arbidol, darunavir, and chloroquine. Thymalfasin and intravenous immunoglobulin were also administered to enhance immunity (Figure 1, lower panel). Throat swabs or stool specimens were collected every 1–3 days throughout his hospital stay and tested with RT-qPCR, and most of them were positive for SARS-CoV-2 RNA until February 15 (23 days after onset) (Figure 1, upper panel). Four days later, the patient was discharged according to the discharge criterion. He had a normal body temperature for at least 3 days, no respiratory symptoms, two consecutively negative results of SARS-CoV-2 RNA (taken 24 h apart), as well as improved lung lesions revealed by chest CT. Home quarantine and health surveillance were subsequently taken by the local center for disease control.

Three days after discharge, the SARS-CoV-2 RNA test again showed positive results in the patient's stool specimens. To eliminate the potential transmission of SARS-CoV-2, the patient was re-admitted to this hospital on February 23rd (day 31 after onset). Physical examination showed (1) normal body temperature (36.7°C); (2) normal heart rate (85 beats/min), normal breathing rate (19 times/ min), and blood pressure (115/78 mmHg); (3) no clinical symptoms of COVID-19. Chest CT indicated that the original lesions in the upper and lower lobes of the right lung were slightly absorbed and reduced compared with the previous one. The patient was discharged on March 1st after two negative SARS-CoV-2 RNA results in both throat swabs and the stool specimens on February 26 and 27. During the second hospitalization, the patient was kept under close observation, but did not receive any treatment.

### Serological Assay of IgG and IgM Specific to Sars-CoV-2

**Figure 2:**
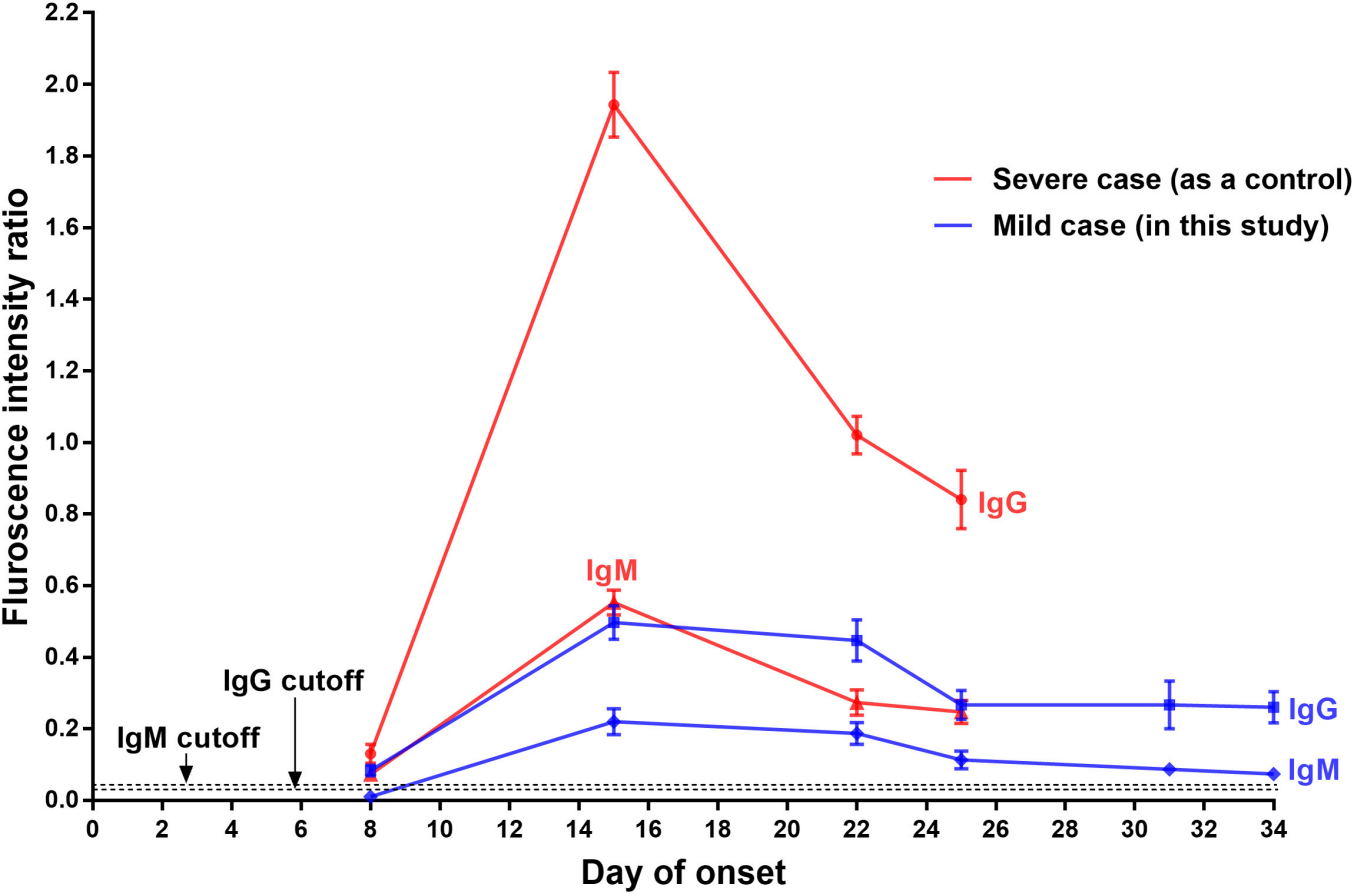
Serological assays of IgG and IgM specific to SARS-CoV-2 of a mild COVID-19 patient, with a severe COVID-19 case as the control. The presence and concentration of IgM and IgG targeting SARS-CoV-2 were measured using a fluorescent immunochromatography detection kit specific to the IgM and IgG against SARS-CoV-2. The cutoff values for IgG and IgM detection were 0.057 and 0.067, respectively.

Serum samples collected from the patient on day 3, 8, 15, 22, 25, 31, and 34 after onset were retrospectively analyzed using a fluorescent immunochromatography detection kit specific to the IgM and IgG antibodies against SARS-CoV-2 (Zhongshan Chuangyi Biochemical Engineering Co.) according to the manufacturer's instructions; the serum of a severe COVID-19 patient (57-year-old, male) collected at the roughly same time was assayed simultaneously as a control. The production of the SARS-CoV-2-specific IgM and IgG in the mild COVID-19 patient showed similar changes: (1) IgM was undetectable and IgG was just a little bit higher than the cutoff value on day 8 of onset; (2) their levels increased equally and seemly peaked on day 15 (with about a 6-fold increase of IgG); (3) both antibodies then decreased and remained relatively stable from day 25 after onset until discharge (Figure 2). In contrast, both the virus-specific IgM and IgG of the control patient were detected at weak levels on day 8 after onset, and then significantly increased, peaking on day 15 (with about a 15-fold increase of IgG). Noticeably, their levels were much higher than those of the mild patient in this report (Figure 2).

## Discussion

A total of 11 COVID-19 patients have been admitted to this hospital so far, and all of them have since been discharged. Compared with other COVID-19 patients in this hospital, the patient in this report had the mildest clinical symptoms but the longest SARS-CoV-2-carrying time (about 30 days from onset of symptoms) and hospitalization time (a total of 35 days). The production of the virus-specific IgM and IgG was remarkably weak, and the high levels lasted for a short time. This mild COVID-19 patient was the only one who was re-admitted to quarantine in this hospital. The reason was a positive SARS-CoV-2 RNA test result in his stool specimen after discharge. Recurrence of positive SARS-CoV-2 RNA in patients who have recovered from COVID-19 has been reported (6–8). This may be explained by: (1) intermittent virus shedding at the end of infection; (2) low viral load and insufficient sensitivity of the kit detection, leading to false negative detection before discharge; and (3) limitation of specimen types and irregular collection (7–9). Of note, this mild COVID-19 patient produced only a small amount of IgM and IgG relative to the strong production of specific antibodies in the control patient, who had severe COVID-19 (Figure 2), and whose virus-carrying time was about 21 days from onset of symptoms (data not shown). We here speculate that low production of the specific antibodies and short maintenance time of high antibody concentration may contribute to the long virus-carrying time in mild cases of COVID-19. A study conducted in 12 severe patients and 11 mild patients reported that the majority of mild patients had no detectable SARS-CoV-2 RNA after 10 days post-onset but showed strong IgG response comparable to that of most severe patients (10), which provides supportive evidence for our speculation.

The immune response to SARS-CoV infection in humans has been studied intensively. The SARS-CoV-specific IgM and IgG antibodies generally begin to appear after 3–42 days and 4–47 days after initial infection; the antibody titers then increase significantly and peak at 13–80 days; the IgM and IgG maintenance time is up to 28–210 days and 90–750 days, respectively (11–16). Our serological assay results suggested that the production characteristics of the IgM and IgG targeting SARS-CoV-2 might differ from those of the antibodies targeting SARS-CoV, particularly in the peak time and duration of the antibodies at high level. This speculation is supported by the findings of newly released publications, which show that the SARS-CoV-2-specific IgM and IgG generally began to appear on day 2–28 and day 2–19 after onset of symptoms, respectively, peaking on day 10–30 after onset (17, 18). Due to one to one case comparisons and lack of continuous daily blood sample collection (Figure 2), more cases, samples and experimental data should be collected and investigated in detail to characterize the relationship between the virus-specific IgM and IgG level change and disease course in mild cases with prolonged SARS-CoV-2 infection.

According to the fifth edition of the National Diagnosis and Treatment Guide for Novel Coronavirus Disease (1), the patient's throat swab or stool sample were monitored for SARS-CoV-2 RNA throughout his hospitalization period. We found a negative result in the throat swab and a positive one in the stool on day 20 after onset; positive results in both types of samples on day 21; however, a positive result in the throat swab and negative result in the stool on day 23 and *vice versa* on day 30 (Figure 1, upper panel). These results suggested that the disease course of COVID-19 is not necessarily related to the SARS-CoV-2 nucleic acid test results in different types of specimens. Intermittent virus shedding could be responsible for the inconsistent viral RNA results in the specimens collected at different time points; however, irregular sample collection should not be excluded. The patient's mother and younger sister were subsequently confirmed to have mild COVID-19 and were admitted to other local hospitals, which suggested that the SARS-CoV-2 virus in this mild case may have low pathogenicity but high infectivity. Longer viral RNA duration does not necessarily mean that an infectious virus is present in clinical specimens; however, live SARS-CoV-2 viruses were found in stool specimens, suggesting a potential transmission through the fecal route (19). Considering that this mild patient lived in a densely populated area, the subsequent re-admission and hospital quarantine were necessary to block the potential virus transmission within the community.

## Conclusion

In conclusion, strict quarantine and health surveillance measures should be taken for all discharged COVID-19 patients due to the potential recurrence of positive SARS-CoV-2 RNA. Weak production and short maintenance of high concentrations of the SARS-CoV-2-specific IgM and IgG may also contribute to the slow virus eradication.

## Data Availability Statement

The raw data supporting the conclusions of this article will be available on request to the corresponding authors, without undue reservation.

## Ethics Statement

Ethical review and approval were not required for the study on human participants in accordance with the local legislation and the requirements of the Ethics Committee of Meizhou People's Hospital. The patient gave written informed consent to publish this case report.

## Author Contributions

XG, ZZho, and LZ were major contributed in writing this manuscript. ZH, ZZho, and XG participated in the diagnosis and treatment of the patient. XG, YH, and ZZha performed the laboratory test. XG performed the design. LZ and YH were involved in the data collection. All authors read and approved the final manuscript.

## Conflict of Interest

The authors declare that the research was conducted in the absence of any commercial or financial relationships that could be construed as a potential conflict of interest.
